# Fast Compressed Sensing of 3D Radial T_1_ Mapping with Different Sparse and Low-Rank Models

**DOI:** 10.3390/jimaging9080151

**Published:** 2023-07-26

**Authors:** Antti Paajanen, Matti Hanhela, Nina Hänninen, Olli Nykänen, Ville Kolehmainen, Mikko J. Nissi

**Affiliations:** Department of Technical Physics, University of Eastern Finland, 70211 Kuopio, Finland; antti.paajanen@uef.fi (A.P.); matti.hanhela@uef.fi (M.H.); nina.hanninen@uef.fi (N.H.); olli.nykanen@uef.fi (O.N.); ville.kolehmainen@uef.fi (V.K.)

**Keywords:** compressed sensing, T_1_ relaxation, quantitative magnetic resonance imaging, regularization, image reconstruction

## Abstract

Knowledge of the relative performance of the well-known sparse and low-rank compressed sensing models with 3D radial quantitative magnetic resonance imaging acquisitions is limited. We use 3D radial T_1_ relaxation time mapping data to compare the total variation, low-rank, and Huber penalty function approaches to regularization to provide insights into the relative performance of these image reconstruction models. Simulation and ex vivo specimen data were used to determine the best compressed sensing model as measured by normalized root mean squared error and structural similarity index. The large-scale compressed sensing models were solved by combining a GPU implementation of a preconditioned primal-dual proximal splitting algorithm to provide high-quality T_1_ maps within a feasible computation time. The model combining spatial total variation and locally low-rank regularization yielded the best performance, followed closely by the model combining spatial and contrast dimension total variation. Computation times ranged from 2 to 113 min, with the low-rank approaches taking the most time. The differences between the compressed sensing models are not necessarily large, but the overall performance is heavily dependent on the imaged object.

## 1. Introduction

Compressed sensing (CS) is one way to speed up magnetic resonance imaging (MRI) by collecting less data while simultaneously retaining high image quality. Essentially, CS theory states that only a small subset of k-space samples is required for accurate image reconstruction [1,2]. In an MRI, CS can be realized by undersampling the k-space incoherently, which produces noise-like artifacts in the image domain. Then, by utilizing sparsifying transforms, compressibility of the MRI images is enforced in the image reconstruction, which minimizes image noise and artifacts, allowing for the original image to be mostly recovered [3,4].

One of the MRI methods most in need of fast acquisition strategies is the 3D quantitative MRI (qMRI) approach. qMRI methods offer extra information that can be valuable in certain situations, but they are also multiple times slower than the standard contrast-weighted MRI. The benefits of 3D imaging include more comprehensive coverage of the anatomy of interest, which in most cases is a welcome addition, but at a further cost to acquisition times. Thus, combining these two time-consuming methods is problematic and necessitates an acceleration of the acquisition methods. Coincidentally, 3D qMRI is a good candidate for CS—undersampling in three dimensions spreads the arising artifacts better when compared to 2D, and the added parametric dimension can be exploited in various ways for better image quality and more aggressive undersampling [5,6,7,8,9,10].

For all CS methods, significant attention should be paid to the undersampling scheme of the acquisition, as it heavily influences how the artifacts manifest in the images and, thus, how well the original image can be recovered [3,11]. Practical considerations and scanner hardware limit the possible trajectories. From the standard k-space sampling schemes, non-cartesian radial trajectories are well suited for accelerated 3D imaging. For radial acquisitions, even for uniform undersampling, the undersampling artifacts are inherently spread out in all three dimensions, and the k-space center is densely sampled, making the acquisition resilient to motion and undersampling.

The downside of the non-cartesian trajectories is that the image reconstruction is computationally much more demanding since the standard fast Fourier transform needs to be replaced by a non-uniform fast Fourier transform (NUFFT) [12]. In addition, usually iterative reconstruction methods are utilized for non-cartesian acquisitions, further increasing the computational cost. Since the large-scale optimization problem of the 3D qMRI CS is usually solved with first-order optimization methods, which require many iterations, the computational aspect of 3D radial qMRI CS needs to be considered. Recently, preconditioned primal-dual proximal splitting was demonstrated to significantly speed up the convergence of CS image reconstructions without the increased noise amplification of the traditional density correction preconditioner approach [13]. The novel preconditioner combined with algorithm implementation on a graphics processing unit (GPU) seems to offer feasible reconstruction times for 3D qMRI CS, negating a large part of the downsides of the non-cartesian CS.

In this study, we approach the topic of CS qMRI by demonstrating the performance of multiple CS models with variable flip angle 3D radial T_1_ mapping. The T_1_ relaxation time, for example, shows promise as a potential biomarker for lung disease [14] and myocardial fibrosis [15]. Additionally, a specific variable flip angle acquisition shows promise in proton resonance frequency shift thermometry [16]. T_1_ mapping has also been combined with compressed sensing to, for example, make single-breath-hold multiparametric MRI feasible [17]. 

The CS models tested include spatial and contrast dimension total variation, spatial and contrast dimension finite differences with the Huber penalty function, and locally low rank and locally low rank combined with spatial total variation. These sparsifying priors are well studied, but their performance with true 3D radial acquisitions is not well defined. The large-scale image reconstruction problems are solved in a feasible time frame with the primal-dual proximal splitting (PDPS) algorithm [18], as it permits preconditioning for faster convergence [13,19] and allows the implementation of the sparsifying priors [20]. Reconstructions are computed with various amounts of data to investigate the undersampling tolerance of the methods. Ex vivo and simulation data are used, as the former provides evidence of real-world performance and the latter gives us the possibility to measure performance with respect to the known ground truth.

## 2. Theory

The standard MRI measurement model is

(1) m=Fu+e, 

where $m\in C^{M}$ is the measured k-space, F is the discrete Fourier transform, $u\in C^{N}$ is the complex-valued image, $N=N_{x}\times N_{y}\times N_{z}$, and $e\in C^{M}$ models the complex-valued measurement noise. With radial acquisitions, the discrete Fourier transform is usually approximated with the non-uniform Fourier transform (NUFFT) operator. Considering also that an image series is acquired, the measurement model can be written as

(2) $$m_{n}=A_{n}u_{n}+e_{n},$$

where the subscript n denotes the contrast index, $A_{n}=P_{n}FC_{n}$ is the NUFFT operator, where $P_{n}$ is the combined interpolation and sampling matrix, and $C_{n}$ is the scaling matrix. Note that the NUFFT operator changes with the contrast, as the sampling trajectories differ between the contrast images.

The optimization problem for the standard non-regularized radial reconstruction then reads

(3) $$\hat{u}=\underset{u}{argmin}\left\{\sum_{n}^{N_{c}}{\left\|A_{n}u_{n}-m_{n}\right\|}_{2}^{2}\;\right\},$$

where u is the 4D image stack containing all $N_{c}$ of the 3D contrast images. This is the least squares solution to the problem and will be referenced as LS for the rest of the publication.

### 2.1. Total Variation Model (sTV + cTV)

One of the most popular regularization functions is the total variation (TV). TV regularizes the L1 norm of the spatial or contrast dimension image gradient, promoting piecewise constancy in the respective dimensions. The performance of TV has been well documented in numerous applications. However, it is plagued by the so-called staircasing effect, where originally smooth image areas are reconstructed as piecewise constant, leading to sub-optimal performance. By utilizing TV in the spatial and contrast dimensions separately, the minimization problem is

(4) $$\hat{u}=\underset{u}{argmin}\left\{\sum_{n}^{N_{c}}\left({\left\|A_{n}u_{n}-m_{n}\right\|}_{2}^{2}\;+\alpha {\left\|\nabla_{s}u_{n}\right\|}_{1}\right)+\beta {\left\|\nabla_{c}u\right\|}_{1}\;\right\},\;$$

where $\nabla_{s}$ is the discrete spatial gradient operator, $\nabla_{c}$ is the discrete contrast dimension gradient operator, and parameters α,β>0 determine the weighting of the spatial and contrast dimension regularization terms, respectively. In our testing, this model is comparable to spatio-temporal TV, where the spatial and contrast image gradients are combined under the same square root, i.e., in the same regularization term.

### 2.2. Huber Model (sH + cH)

The Huber penalty function is of the form

(5) $$H_{\gamma }\left(x\right)=\left\{\begin{array}{c} \frac{{\left|x\right|}^{2}}{2\gamma },\;\;\;\left|x\right|\leq \gamma ,\\ \left|x\right|-\frac{\gamma }{2},\;\;\;\left|x\right|>\gamma , \end{array}\right.$$

where γ is the tunable Huber parameter. Below γ parameter, the Huber regularization is close to the standard L2-norm smoothness regularization, and above γ, it approximates the TV regularization. In the context of MRI, the γ parameter can be interpreted as a threshold where discontinuous signal change is assumed [21]. The Huber penalty function has been proposed as a remedy to the stair-casing of TV in the denoising context [18], and it has been applied, e.g., as a temporal regularization for DCE data [21].

The image reconstruction problem utilizing the Huber penalty function on the spatial and contrast dimension image gradients is

(6) $$\hat{u}=\underset{u}{argmin}\left\{\sum_{n}^{N_{c}}\begin{array}{c} \left({\left\|A_{n}u_{n}-m_{n}\right\|}_{2}^{2}+{\alpha \left\|H_{\gamma_{1}}\left(\nabla_{s}u_{n}\right)\right\|}_{1}\right)\\ +{\beta \left\|H_{\gamma_{2}}\left(\nabla_{c}u\right)\right\|}_{1} \end{array}\;\right\}.$$

The two separate Huber parameters, $\gamma_{1}$ and $\gamma_{2}$, provide a way to spatially alleviate the piecewise constant nature of TV while also limiting excessive contrast dimension smoothness. Contrast dimension smoothness is not a desirable property with a qMRI image series because the resulting temporal blur can lose small nuances of the contrast modulation, and the stair-casing effect is not as significant an issue temporally as the images undergo the signal fitting procedure.

### 2.3. Locally Low-Rank Model (LLR)

A low-rank model assumes that for the $N_{x}N_{y}N_{z}\times N_{c}$ Casorati matrix, formed of the $N_{x}\times N_{y}\times N_{z}\times N_{c}$ qMRI image series, the rank of the Casorati matrix is much smaller than $N_{c}$, i.e., it is rank deficient. It has been shown that this rank deficiency can be improved if only a small part of the image series that contains mostly similar signal dynamics is considered. This approach is known as the locally low-rank (LLR) model [22]. The superior performance of the LLR model when compared to the (globally) low-rank model has been, for example, demonstrated with DCE MRI [23] and quantitative parameter mapping of T_1_ and T_2_ relaxation times [9]. If the low-rank assumption does not hold and low-rank regularization is enforced during image reconstruction, contrast dimension blurring or unsatisfying noise suppression will occur, depending on the strength of the regularization [22].

Rank-constrained optimization problems are usually infeasible in practice, but luckily, convex relaxations that utilize nuclear norm regularization can be used [24]. Thus, the LLR optimization problem can be written as

(7) $$\hat{u}=\underset{u}{argmin}\left\{\sum_{n}^{N_{c}}{\left\|A_{n}u_{n}-m_{n}\right\|}_{2}^{2}+\alpha \sum_{b\in \Omega }{\left\|C_{b}u\right\|}_{*}\;\right\},$$

where ${\left\|\;.\right\|}_{*}$ denotes the nuclear norm, $C_{b}$ is an operator that extracts an image block of size $b\times b\times b\times N_{c}$ from the image series and reshapes it to a Casorati matrix of size $b^{3}\times N_{c}$, and Ω denotes the whole set of image blocks. The blocks can be chosen as overlapping or non-overlapping, with the computational complexity increasing as a function of the overlap. Translational invariance can be achieved by randomly spatially shifting the contrast images prior to LLR regularization at each iteration during the reconstruction [25].

### 2.4. Spatial Total Variation and Locally Low-Rank Model (sTV + LLR)

Adding spatial TV regularization to an LLR model can provide multiple benefits [26,27,28]. If the qMRI series is not exactly low rank, the spatial TV regularization can be used to suppress the left-over noise. Additionally, spatial TV can also be used to minimize the blocking artifacts that the LLR might induce. The joint optimization problem then reads

(8) $$\hat{u}=\underset{u}{argmin}\left\{\begin{array}{c} \sum_{n}^{N_{c}}\left({\left\|A_{n}u_{n}-m_{n}\right\|}_{2}^{2}\;+\alpha {\left\|\nabla_{s}u_{n}\right\|}_{1}\right)\\ +\beta \sum_{b\in \Omega }{\left\|C_{b}u\right\|}_{*}\; \end{array}\right\}.$$

### 2.5. Primal-Dual Proximal Splitting Algorithm

All the models detailed above can be solved by the PDPS algorithm [18]. The PDPS solves an optimization problem simultaneously with its dual and can be applied to primal minimization problems of the form

(9) $$\underset{x}{\min}F\left(Kx\right)+G\left(x\right),$$

where K is a linear transform, and F and G are convex functions. For Equation (9), the PDPS algorithm reads (Algorithm 1) [20].
**Algorithm 1.** Primal-dual proximal splitting.Choose θ∈[0,1] and σ,τ such that $\sigma \tau <1/{\left\|K\right\|}_{2}^{2}$
**while** not converged **do**  $y^{k+1}≔prox_{\sigma }[F^{*}](y^{k}+\sigma K{\bar{x}}^{k})$
  $x^{k+1}≔prox_{\tau }[G](x^{k}+\tau K^{T}y^{k+1})$
  ${\bar{x}}^{k+1}≔x^{k+1}+\theta (x^{k+1}-x^{k})$
**end while**

Where prox denotes the proximal mapping, which is used to generate the descent direction for the function argument.

As an example, for Equation (3) and a single-contrast image, the optimization problem can be written as in Equation (9) by setting the primal variable x as the vectorized complex image u, K as the corresponding NUFFT operator A, $F\left(p\right)={0.5\left\|p-m\right\|}_{2}^{2}$, where p=Au, and $G\left(x\right)=0$. The proximal mapping of the convex conjugate of F is $prox_{\sigma }\left[F^{*}\right]\left(y\right)=(y-\sigma m)/(1+\sigma ).$ Additionally, we let the algorithm utilize a diagonal preconditioner P for the dual variable following [13]. With these, the preconditioned PDPS algorithm reads (Algorithm 2).
**Algorithm 2.** Preconditioned primal-dual proximal splitting for least squares.Choose θ∈[0,1] and σ,τ such that $\sigma \tau <1/{\left\|A^{T}PA\right\|}_{2}^{2}$
**while** not converged **do**  $p^{k+1}≔(p^{k}+P\sigma (A{\bar{u}}^{k}-m))/(1+P\sigma )$
  $u^{k+1}≔u^{k}-\tau A^{T}p^{k+1}$
  ${\bar{u}}^{k+1}≔u^{k+1}+\theta (u^{k+1}-u^{k})$
**end while**

The PDPS implementations for Equations (4) and (6)–(8) are detailed in the Appendix A (Algorithms A1–A4). For further information about the algorithm, the reader is instructed, e.g., to the references [18,20].

### 2.6. Variable Flip Angle Acquisition

T_1_ mapping can be performed by a variable flip angle (VFA) acquisition, where multiple images with different flip angles (FA) but with the same repetition time (TR) are acquired from which the T_1_ value can be solved. It is possible to estimate the T_1_ from only two flip angles [29]. However, this estimation is accurate only for a very limited range of T_1_s and, thus, the use of multiple flip angles allows for a more robust estimation [30]. 

For a SWIFT-type acquisition, where the T_2_* effects can be neglected and the signal is acquired in a steady state [31], the signal equation is

(10) $$u_{n}=S_{0}\frac{\left(1-\exp\left(-TR/T_{1}\right)\right)\sin\left(FA_{n}\right)}{1-\exp\left(-TR/T_{1}\right)\cos\left(FA_{n}\right)},\;$$

where S_0_ is the proton density and $n=1,\ldots ,N_{c}$. The T_1_ and S_0_ values can be solved by either linearizing the signal equation [29,32] or by performing non-linear least squares fitting.

## 3. Methods

The image reconstruction is performed with the preconditioned PDPS algorithm detailed in the previous section and in the Appendix A. Each image reconstruction problem, Equations (3), (4), and (6)–(8), is evaluated on two radial datasets: a VFA Multi-Band-Sweep Imaging with Fourier Transform (MB-SWIFT) [33] ex vivo acquisition of a mouse spine and a simulated phantom. The ex vivo specimen allows us to measure real-world performance of the models, and the phantom gives a proper ground truth reference. We detail the two datasets and the image reconstruction specifics below.

In the spirit of open and reproducible research, both datasets, the Python codes for image reconstruction, and the Matlab codes for signal fitting are available from Zenodo (https://doi.org/10.5281/zenodo.8177285).

### 3.1. Ex Vivo Multi-Band-SWIFT Acquisition

A section of a mouse spine with the surrounding tissues was collected from a mouse that was sacrificed according to ethical permits (for an unrelated study ESAVI/270/ 04.10.07/2017 [34]) and subsequently transcardially perfused, fixed, and stored with 4% paraformaldehyde. For imaging, the sample was inserted into a 3D-printed holder and immersed in perfluoropolyether (Galden HS240, Vacuumservice Oy, Helsinki, Finland).

A VFA acquisition with the MB-SWIFT was performed with a small-bore 9.4 T Varian/Agilent MRI scanner (VnmrJ DirectDrive software v.3.2, Varian Associates Inc., Palo Alto, CA, USA) and a 19 mm diameter quadrature radiofrequency volume transceiver (Rapid Biomedical, Rimpar, Germany). The magnet shimming was performed manually. The essential MB-SWIFT parameters were a bandwidth of 385 kHz, an isotropic field of view of 3 cm, 63488 radial spokes per contrast, an image size of $256^{3}$, and flip angles of 1, 2, 3, 4, 5, 6, 7, 8, 10, 12, 14, and 20 degrees. The standard MB-SWIFT data processing pipeline [33] was followed until a radial k-space was acquired, wherefrom all subsequent data processing was performed with the codes developed here and shared in the Zenodo archive.

Our VFA implementation utilized unique gradient trajectories for the acquisition of each contrast. This choice was made to achieve sampling where the image noise and undersampling artifacts would be incoherent, to a degree, between images, thus allowing contrast dimension regularization to effectively eliminate these in the image reconstruction. Specifically, each contrast acquisition utilized 128 unique and complementary gradient trajectories, each consisting of 512 center-out spokes. All these trajectories filled the k-space uniformly, but none of them were identical. Retrospective downsampling, mimicking a sparse acquisition, was completed using a part of the trajectories in the reconstruction, making the downsampling readily transformable to practice. Imaging time for the whole VFA series was 22 min and 24 s.

### 3.2. Simulated Phantom

**Figure 1:**
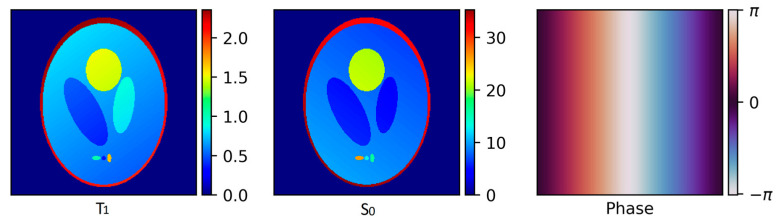
The simulated phantom.

For the simulated phantom, separate S_0_ and T_1_ volumes were created [35]. For the phase, a simple linear ramp from zero to zero with a wrap in the middle was used (Figure 1). The gradient trajectories, flip angles, and repetition time for the data simulation were taken from the MB-SWIFT acquisition. The complex image series was generated with (10) and the phase-component, and non-uniform discrete Fourier transform was used to generate the complex radial k-space. Complex Gaussian white noise with standard deviation of two percent of the mean absolute noiseless k-space was added to the simulated k-space data.

### 3.3. Error Metrics

The performance of the CS image reconstruction was evaluated with the normalized root mean squared error (nRMSE) and structural similarity index (SSIM) [36] in a manually segmented region of interest covering the whole specimen. nRMSE is defined by $nRMSE(x)={\left\|x-x_{ref}\right\|}_{2}/{\left\|x_{ref}\right\|}_{2}$, where $x_{ref}$ is the ground truth. Obviously, ground truth data are only available for the simulation. Thus, for the ex vivo data, for each model, the reference T_1_ and S_0_ maps were formed by performing non-linear least squares fitting on image series reconstructed from the full data with slight regularization. Regularization for the reference reconstructions is important, as even fully sampled radial reconstruction can suffer from increased noise and undersampling artifacts [37]. Our approach, thus, visualizes the relative tolerance to undersampling for each method for the ex vivo data. The simulation data then describe each model’s absolute tolerance to undersampling.

### 3.4. Computation

The image reconstruction was performed by solving Equations (3), (4), and (6)–(8) with the preconditioned PDPS algorithm. The data used in the reconstruction are presented with acceleration factor (AF), which is defined as (#full data)/(#data used in the reconstruction). Our definition of full data reconstruction is close to that of a corresponding cartesian acquisition, i.e., our full data reconstruction is already accelerated by about a factor of pi. Since the amount of data acquired during imaging is almost directly proportional to imaging time, AF mirrors the proportional imaging time speed up. 

The preconditioner was calculated for each contrast image prior to the PDPS reconstruction. The step-size scheme for the PDPS algorithm was adapted from [13] as we set σ=1 and $\tau =1/{\left\|K^{T}PK\right\|}_{2}$, where the operator norm was calculated with the power method. Each reconstruction was continued for a maximum of 500 iterations or until relative difference of 20 objective function values less than $10^{-2}$ for the ex vivo data or $10^{-3}$ for the phantom data was reached. All the optimal reconstructions, regularization-wise, stopped due to the stop criterion. Different stopping tolerances were used because of the possible slight k-coordinate inaccuracies and higher noise level of the ex vivo data. The optimal regularization parameters were chosen with a grid search by minimizing T_1_ nRMSE against the respective reference reconstruction. For the grid search, linearized T_1_ fitting was completed for every reconstruction to obtain quick estimate for the T_1_. Once optimal reconstruction was found, the T_1_ and S_0_ maps of the optimal reconstruction were updated to the non-linear least squares fit estimates.

## 4. Results

**Figure 2:**
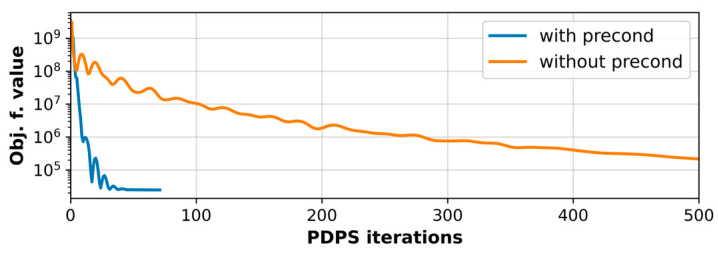
Convergence of Equation (3) with and without utilizing the preconditioner [13] in the reconstruction. The preconditioned PDPS algorithm convergences significantly faster.

The significantly faster convergence of the preconditioned PDPS algorithm was verified and is visualized in Figure 2, where the objective function value of Equation (3) for fully sampled simulation data reconstruction is plotted. With the preconditioner, the PDPS algorithm reached the stop criterion in 72 iterations. The non-preconditioned variant that utilized step sizes of $\sigma =\tau =1/\sqrt{{\left\|K\right\|}_{2}}$ [18,20] ran 5000 iterations (500 shown in the image) and did not achieve the same objective function value as the preconditioned version. Similar gains in convergence were achieved for all the reconstruction models. Slight oscillations that are visible in the objective function value were largely unaffected by the choice of the step sizes.

**Figure 3:**
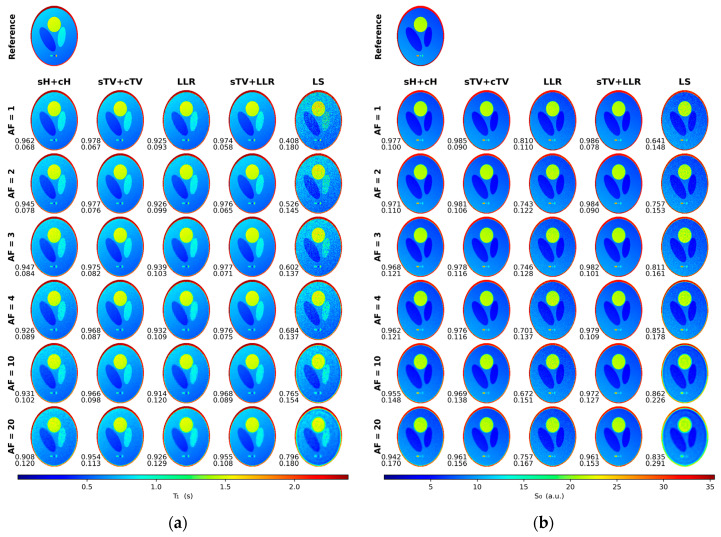
The estimated T_1_ (**a**) and S_0_ (**b**) maps for the multiple tested acceleration factors (AF) for the simulated phantom. The lower left corner of each image shows the SSIM (**top**) and nRMSE (**bottom**) values of the respective reconstruction.

**Figure 4:**
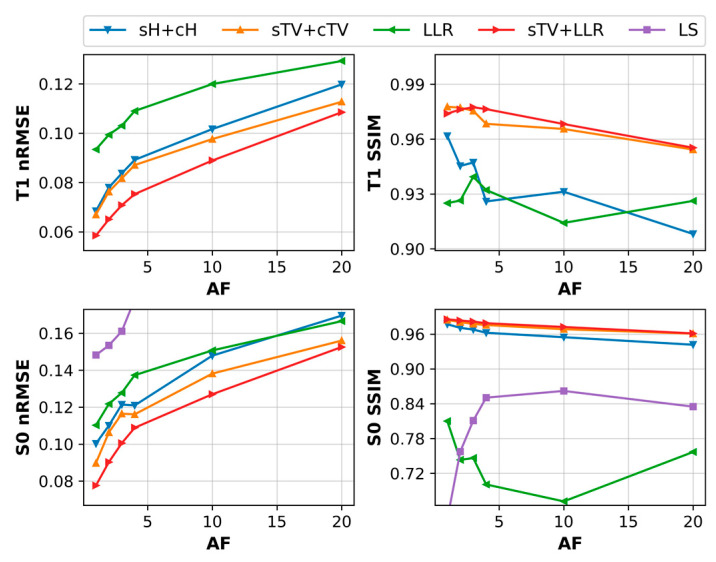
The T_1_ and S_0_ map error metrics as a function of AF for the simulated phantom.

With the simulation data, we observed good tolerance for undersampling for all the regularized models. T_1_ maps, with AFs up to 20, remain of high image quality. The sTV + cTV and sTV + LLR models outperform every other method, with the latter achieving the lowest nRMSE scores while being almost identical in performance SSIM-wise. Staircasing is increasingly visible as AF grows, with the sTV + LLR model generally suffering less from it. The sH + cH model clearly leaves more noise in the reconstructions and subsequently performs slightly worse nRMSE-wise and a lot worse SSIM-wise when compared to the TV model. The LLR model demonstrates some residual noise in areas where the blocks overlap multiple image details, e.g., around the outer ring of the phantom, and the reconstructions also look over-regularized with faint block artifacts (Figure 3a and Figure 4).

Generally, for the S_0_ component, the small details on the phantom are visible for all models for all Afs but with varying amounts of blur, and the nRMSE values are overall larger than with the T_1_ maps. The sTV + LLR model is again the best model, with the sTV + cTV model a close second, as agreed by both error metrics. The sH + cH model suffers from image blur the most and is the second-worst model here. The LLR model has the most noise and thus the highest nRMSE and the worst SSIM values (Figure 3b and Figure 4).

**Figure 5:**
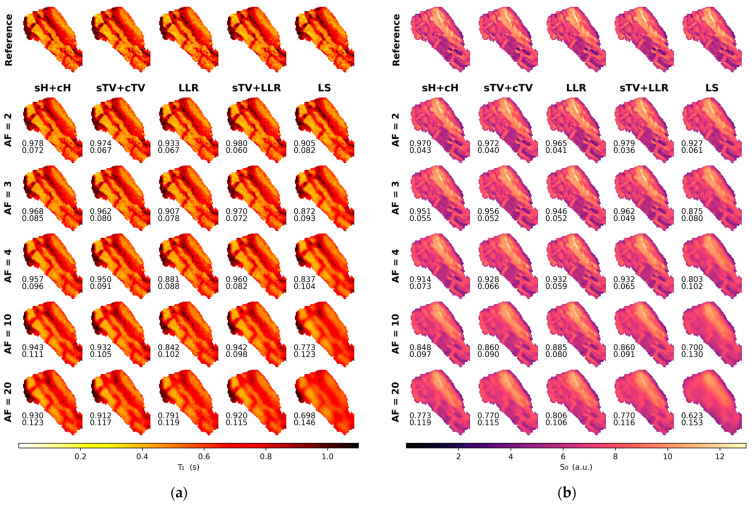
The estimated T_1_ (**a**) and S_0_ (**b**) maps for the multiple tested acceleration factors (AF) for the ex vivo acquisition. The lower left corner of each image shows the SSIM (**top**) and nRMSE (**bottom**) values of the respective reconstruction.

**Figure 6:**
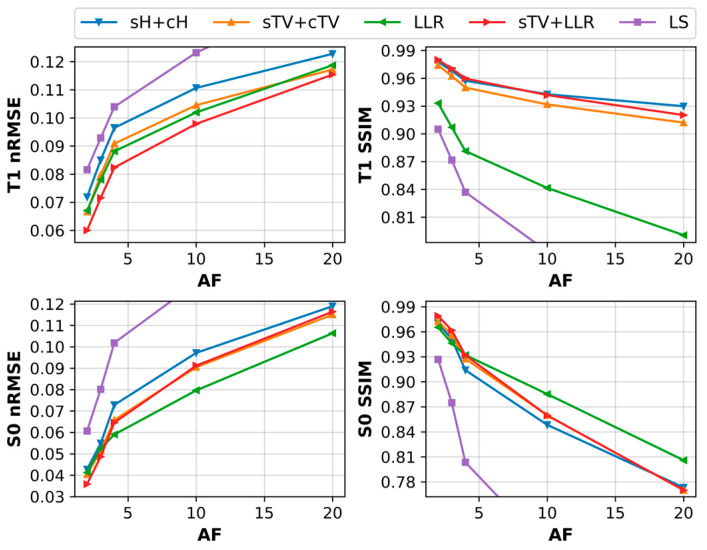
The T_1_ and S_0_ map error metrics as a function of AF for the ex vivo acquisition.

The ex vivo data do not tolerate undersampling as well as the phantom data. For all regularized models, T_1_ maps with Afs up to four remain visually similar and under 10% in nRMSE when compared to the respective reference. Afs larger than four suffer from significant image blur, and image details are consequently lost. This is clearly visible in the space between the vertebra, where the areas with high T_1_ values are lost. All the regularized models are relatively close in performance, with the sTV + LLR outperforming others for all Afs tested nRMSE-wise and being the optimal model for the Afs up to four SSIM-wise. The LLR model outperforms the sH + cH and sTV + cTV models in nRMSE, but the reconstructions are clearly noisier, and the SSIM suffers greatly. The SSIM favors the Huber model as it outperforms the LLR and sTV + cTV models for all Afs. Additionally, the Huber model is the optimal one, SSIM-wise, for Afs of 10 and 20. Note that the error metrics are calculated with respect to the corresponding full-data reference reconstructions (Figure 5a and Figure 6).

For Afs of four and below, all the reconstruction models perform similarly and produce low nRMSE and high SSIM values for the S_0_-component, but further data reduction quickly deteriorates the image quality. The sTV + LLR model is the best below fourfold acceleration by a small margin in both error metrics. However, the LLR model retains the most image details and noise as AF increases, while all others suffer from significant image blur (Figure 5b and Figure 6).

## 5. Discussion

We demonstrated the performance of multiple CS image reconstruction models in a qMRI setting and how the preconditioned PDPS algorithm, relying additionally on fast GPU implementation, allows for a computationally fast solution of the chosen models. Without the preconditioner, computation times for the reconstructions would have been prohibitively long, even with the GPU implementation. By nRMSE and SSIM of the T_1_ maps, generally the best-performing model was the sTV + LLR for both phantom and specimen data. However, the differences between all the tested models are not necessarily large.

The similar performance of the models is not very surprising, as they do promote sparsity in a reasonably similar manner. For the models utilizing contrast dimensions TV and Huber, the minimum solution, regularization-wise, would be an image series of constant contrast, as the contrast differences would then be zero. Similarly, for the low-rank model, the minimum solution would be a rank-one matrix, meaning that the matrix can be represented as a matrix product between a contrast dimension vector and a spatial dimension vector (vectorized image). That is, the minimum solution is a matrix where only specific (though not necessarily constant) signal evolution is allowed in the contrast dimension. For locally low rank, this holds block-wise. These slight fundamental differences seem not to significantly affect the performance of the models.

The best-performing model was the sTV + LLR, with the TV model being a close second, indicating that LLR regularization is slightly better than cTV for the VFA acquisition. The combination of large (from a flip angle of one to two) and small (from a flip angle of seven to eight) signal jumps is known to be non-optimal for TV. Thus, discarding the flip angle of one from the acquisition might close this performance gap.

The Huber model was tuned so that most of the small signal changes were below the Huber parameter value, i.e., flat image areas would be reconstructed piecewise-smooth. However, here the models are compared after a non-linear fitting procedure, and the effect of the Huber penalty function is mostly seen as increased noise in the phantom T_1_ maps. A positive feature of the Huber model is that it also permits classical gradient-based reconstruction methods (e.g., conjugate gradient) to be used, but in our case, the loss of the preconditioner with the classical methods makes these still slower than the PDPS. However, as the Huber and TV models were close in performance, Huber could still be an interesting choice for cartesian reconstructions where no preconditioner is needed.

With the LLR model, optimal regularization would threshold out the singular vectors corresponding to pure noise, with over-regularization then affecting the signal evolution itself and under-regularization leading to residual noise. Thus, by under-regularizing, noise can be selectively retained without imposing strict a priori information (i.e., piecewise smoothness) on the image estimate, which might explain why the LLR model performs well with the noisy ex vivo specimen. For the phantom data, the LLR minimum nRMSE T_1_ maps suffer from block artifacts, which could be alleviated with 50% block overlap, with substantial increases in computation time or with an sTV as shown. The block artifacts are also amplified in the signal fitting, as no clear artifacts are visible on the magnitude images (not shown here).

The model combining spatial TV and LLR alleviates the block artifacts and further suppresses the leftover noise of the LLR regularization, making it the best model with the simulation data. With the ex vivo specimen, the sTV + LLR is not a significant improvement over the LLR model, and the LLR reconstructions can retain even more image details, as is particularly visible in the S_0_ maps. This behavior can probably be admitted to the noise and more complex structure of the reference reconstruction since, with the simulation data, the sTV + LLR clearly outperform the LLR model.

In this study, we are missing one widely acknowledged regularization model, the total generalized variation (TGV) [38]. TGV was not included because the memory footprint of the model is much larger than that of the TV approach. The larger memory footprint prevented us from doing the reconstruction using the GPU, as all other reconstructions were completed, and the CPU implementation would have been infeasibly slow. As the 3D TV demonstrated some staircasing artifacts, it is a shame that no comparison to TGV could be performed, as minimizing the staircasing artifacts is what TGV was originally proposed for. 

Ex vivo data were utilized to tie the performance of the tested models to a more realistic case compared to the ideal phantom data. Although the ex vivo data cannot be used to address the possible motion and complications of multiple coils of clinical in vivo data, the ex vivo data at least introduce slight k-coordinate inconsistencies that are inherent in radial acquisitions. Additionally, the noise might also differ slightly from the ideal assumed complex white Gaussian noise, as in ex vivo acquisition, there are multiple possible sources of noise and artifacts, such as eddy currents.

The comparison of the methods between the simulation and ex vivo data is not straightforward because of the lack of real ground truth for the ex vivo specimen. However, the ex vivo specimen can be thought to be the more “correct” comparison, as it represents the image that we are usually trying to match with CS methods. With our ex vivo specimen, the reference happens to be quite noisy, which opens up the topic that the fully sampled reference could also be improved with stronger regularization (suppressing more noise). Doing this would likely increase the performance of the tested models, as the CS methods inherently denoise images. Thus, trying to optimize denoised CS reconstructions for a noisy reference with error metrics that have their own problems does impose some biases. However, as it is unknown which parts of the (reference) image are noise and which are actual details, and further considering that the regularizations impose a priori information on the reconstruction, tuning the optimal reference reconstruction is not clear. Here, we thus chose to use the noisy reference. 

Clear differences between the behavior of nRMSE and SSIM error metrics were observed. Especially SSIM seems to greatly punish the presence of any image noise, even if, to the eye of the reader, the slightly noisy reconstruction looks more “natural”. nRMSE, on the other hand, does not discriminate where the error comes from, i.e., whether it is image noise or loss of detail, and just measures the global error. Using the lowest nRMSE of the T_1_ maps seemed to produce images that matched with the expectation of natural-looking reconstructions; hence, that criterion was used. If the same was completed with SSIM, the individual contrast images and the T_1_ maps looked over-regularized.

We chose not to report any computation time metrics in the results, as the absolute performance is greatly related to the hardware used and implementation details. We note here that for our Python implementation, the reconstruction times in minutes on a computational server with an RTX A6000 GPU ranged between 2.2 and 13.9; 2.3 and 14.3; 26.4 and 111.5; 28.0 and 113.5; and 0.9 and 7.7 for the sTV + cTV, sH + cH, LLR, sTV + LLR, and LS models, respectively. The models utilizing LLR regularization are considerably slower than the rest, as multiple singular value decompositions are needed per iteration. Still, from the reconstruction times, it can be concluded that, with the preconditioner, the reconstruction times are feasible for the large-scale radial 3D qMRI problem. This still ignores the problem of how to set the regularization parameters without a reference reconstruction, which remains an issue for most models. There are some methods that utilize automatic or data-driven regularization parameters, for example, with the wavelet regularization [39] and with TV regularization [40], but to our knowledge, no such methods exist for the models tested here in the qMRI setting.

Lastly, with the chosen VFA acquisition, the ex vivo results are affected by the uniformity of the B_1_ + field, which we did not correct in any way. However, it does not affect the results error-metric-wise since the reference contains the same error. 

## 6. Conclusions

The performance of the tested models varied depending on the dataset and error metric used, but the differences between models were not necessarily large. By T_1_ map nRMSE and SSIM, the best-performing model was sTV + LLR, with sTV + cTV generally being a close second. The latter model might even be preferred due to the much shorter computation time. The Huber model did remedy the staircasing artifact at the cost of increased T_1_ map noise, and, overall, the performance of the Huber model was inferior to the sTV + cTV. The significantly faster convergence of the preconditioned PDPS algorithm with 3D radial data was also verified.

## Data Availability

In the spirit of open and reproducible research, both datasets, the Python codes for image reconstruction, and the Matlab codes for signal fitting are available from Zenodo (https://doi.org/10.5281/zenodo.8177285).

## Appendix A

The pseudocodes for the reconstruction algorithms are detailed below (Algorithms A1–A4). Here, all the necessary proximal mappings have been explicitly written out. The gradient operator and its transpose are set as $\nabla^{T}=-div$, with the subscript denoting the dimensionality of the operation. The $\left|x\right|$ operator denotes the magnitude of a vector-valued image x. Operator $bSVT_{\alpha }(x)$ exhibits singular value soft thresholding to the block Casorati matrices extracted from the image series.

**Algorithm A1.** Preconditioned primal-dual proximal splitting for the TV model of Equation (4).
Choose θ∈[0,1] and σ,τ such that $\sigma \tau <1/{\left\|K^{T}PK\right\|}_{2}^{2}$
**while** not converged **do**
$p^{k+1}≔(p^{k}+P\sigma (A{\bar{u}}^{k}-m))/(1+P\sigma )$
$q^{k+1}≔\alpha (q^{k}+\sigma \nabla_{s}{\bar{u}}^{k})/\max(\alpha ,\left|q^{k}+\sigma \nabla_{s}{\bar{u}}^{k}\right|)$
$r^{k+1}≔\beta (r^{k}+\sigma \nabla_{c}{\bar{u}}^{k})/\max(\beta ,\left|r^{k}+\sigma \nabla_{c}{\bar{u}}^{k}\right|)$
$u^{k+1}≔u^{k}-\tau A^{T}p^{k+1}+\tau div_{s}q^{k+1}+\tau div_{c}r^{k+1}\;$
${\bar{u}}^{k+1}≔u^{k+1}+\theta (u^{k+1}-u^{k})$
**end while**

**Algorithm A2.** Preconditioned primal-dual proximal splitting for the Huber model of Equation (6).
Choose θ∈[0,1] and σ,τ such that $\sigma \tau <1/{\left\|K^{T}PK\right\|}_{2}^{2}$
**while** not converged **do**
$p^{k+1}≔(p^{k}+P\sigma (A{\bar{u}}^{k}-m))/(1+P\sigma )$
$q^{k+1}≔\alpha ((q^{k}+\sigma \nabla_{s}{\bar{u}}^{k})/(1+\sigma \gamma_{1}/\alpha ))/\max(\alpha ,\left|\left((q^{k}+\sigma \nabla_{s}{\bar{u}}^{k})/(1+\sigma \gamma_{1}/\alpha )\right)\right|)$
$r^{k+1}≔\beta ((r^{k}+\sigma \nabla_{c}{\bar{u}}^{k})/(1+\sigma \gamma_{2}/\beta ))/\max(\beta ,\left|\left((r^{k}+\sigma \nabla_{c}{\bar{u}}^{k})/(1+\sigma \gamma_{2}/\beta )\right)\right|)\;$
$u^{k+1}≔u^{k}-\tau A^{T}p^{k+1}+\tau div_{s}q^{k+1}+\tau div_{c}r^{k+1}\;$
${\bar{u}}^{k+1}≔u^{k+1}+\theta (u^{k+1}-u^{k})$
**end while**

**Algorithm A3.** Preconditioned primal-dual proximal splitting for the locally low-rank model of Equation (7).
Choose θ∈[0,1] and σ,τ $\;\mathrm{such}\;\mathrm{that}\;\sigma \tau <1/{\left\|K^{T}PK\right\|}_{2}^{2}$
**while** not converged **do**
$\;p^{k+1}≔(p^{k}+P\sigma (A{\bar{u}}^{k}-m))/(1+P\sigma )$
$\;u^{k+1}≔{bSVT_{\alpha }(u}^{k}-\tau A^{T}p^{k+1})$
$\;{\bar{u}}^{k+1}≔u^{k+1}+\theta (u^{k+1}-u^{k})$
**end while**

**Algorithm A4.** Preconditioned primal-dual proximal splitting for the spatial total variation combined with locally low-rank model of Equation (8).
Choose θ∈[0,1] and σ,τ $\;\mathrm{such}\;\mathrm{that}\;\sigma \tau <1/{\left\|K^{T}PK\right\|}_{2}^{2}$
**while** not converged **do**
$\;p^{k+1}≔(p^{k}+P\sigma (A{\bar{u}}^{k}-m))/(1+P\sigma )$
$\;q^{k+1}≔\alpha (q^{k}+\sigma \nabla_{s}{\bar{u}}^{k})/\max(\alpha ,\left|q^{k}+\sigma \nabla_{s}{\bar{u}}^{k}\right|)$
$\;u^{k+1}≔{bSVT_{\beta }(u}^{k}-\tau A^{T}p^{k+1}+\tau div_{s}q^{k+1})$
$\;{\bar{u}}^{k+1}≔u^{k+1}+\theta (u^{k+1}-u^{k})$
**end while**
